# MiR-193b-3p and miR-132-3p as prognostic biomarkers of survival in pleural mesothelioma patients treated with first-line bevacizumab plus pemetrexed-platinum chemotherapy in the IFCT-0701 MAPS phase 3 trial

**DOI:** 10.1016/j.tranon.2025.102520

**Published:** 2025-09-05

**Authors:** Guénaëlle Levallet, Christian Creveuil, Alexandre Léger-Vigot, Solenn Brosseau, Claire Danel, Arnaud Scherpereel, Sylvie Lantuejoul, Julien Mazières, Laurent Greillier, Clarisse Audigier-Valette, Emmanuel Bergot, Denis Moro-Sibilot, Olivier Molinier, Hervé Léna, Isabelle Monnet, Franck Morin, Valérie Gounant, Gérard Zalcman

**Affiliations:** a Université de Caen Normandie, CNRS, Normandie Université, ISTCT UMR6030, GIP CYCERON, F-14000 Caen, France; b Centre Hospitalier Universitaire de Caen, Service d’Anatomie et Cytologie pathologiques, 14000 CAEN, France; c Biostatistics and Clinical Research Unit, Caen University Hospital, Caen, France; d Université de Caen Normandie, INSERM, Normandie Université, ANTICIPE U1086 INSERM, Caen, France; e Université Paris Cité, Thoracic Oncology Department & CIC1425, Hôpital Bichat-Claude Bernard, Assistance Publique Hôpitaux de Paris (AP-HP), Paris, France; f U830 INSERM "Cancer, Heterogeneity, Instability, Plasticity, A.R.T group", Curie Institute, Paris, France; g Université Paris Cité Department of Pathology, Hôpital Bichat-Claude Bernard, AP-HP, Paris, France; h Department of Pulmonary and Thoracic Oncology, Centre Hospitalier Universitaire Lille, University of Lille, U1019 INSERM, Center of Infection and Immunity of Lille, Lille, France; i Department of Biopathology, Reference National Center MESOPATH, Centre Léon Bérard, Lyon, Grenoble Alpes University, France; j Department of Pulmonology, Hôpital Larrey, University Hospital of Toulouse**,** Toulouse, France; k Department of Multidisciplinary Oncology and Therapeutic Innovations, Assistance Publique Hôpitaux de Marseille, Aix Marseille University, Marseille, France; l Department of Pulmonology, Centre Hospitalier Toulon Sainte-Musse, Toulon, France; m Centre Hospitalier Universitaire de Caen, Service de Pneumologie et Oncologie Thoracique, 14000 CAEN, France; n Pôle Thorax et Vaisseaux, Centre Hospitalier Universitaire Grenoble, Grenoble, France; o Department of Pulmonology, Centre Hospitalier Le Mans, Le Mans, France; p Department of Pulmonology, Ponchaillou University Hospital, Rennes, France; q Department of Pulmonology, CHI Créteil, Créteil, France; r Intergroupe Francophone de Cancérologie Thoracique (IFCT), Paris, France; s Pulmonology and Thoracic oncology Department, Tenon University Hospital, Assistance Publique Hôpitaux de Paris (AP-HP), Paris, France

**Keywords:** Pleural mesothelioma, Bevacizumab, microRNA, Predictive biomarker

## Abstract

We investigated whether angiogenesis-related microRNAs (miRNAs) predict survival in patients with pleural mesothelioma (PM) treated with bevacizumab plus pemetrexed-platinum chemotherapy in the Mesothelioma Avastin Cisplatin Pemetrexed Study ('MAPS', NCT00651456) phase 3 trial phase III trial (NCT00651456). Twelve miRNAs were measured in FFPE samples from 236 of the 448 MAPS trial patients (50.8 %), normalized to RNU48. Overall survival (OS) and progression-free survival (PFS) were analyzed by miRNA expression using univariate and multivariate models adjusted for clinical covariates. Internal validation was performed by bootstrapping. Interaction tests assessed the predictive value of each miRNA with respect to treatment arm. Low miR-193b-3p expression was associated with longer OS in PM patients, as shown in both univariate and multivariate analyses (adjusted HR = 0.87 [0.81–0.93], p < 0.001; bootstrap inclusion fraction [BIF]: 81.3 %), with both treatment arms analyzed together. It also predicted longer PFS (adjusted HR = 0.91 [0.85–0.97], p = 0.0042). Interaction tests revealed that for four miRNAs (miR-155–5p, miR-29c-5p, miR-132–3p, and miR-100–5p), lower expression levels were associated with greater efficacy of the bevacizumab/cisplatin/pemetrexed combination. Notably, the interaction between treatment arms and miR-132–3p expression was statistically significant (p = 0.004). In the IFCT-GFPC-0701 MAPS trial, low miR-193b-3p expression demonstrated significant independent prognostic value, being associated with longer OS and PFS. Additionally, low expression of miR-155–5p, miR-29c-5p, miR-132–3p, and miR-100–5p showed independent predictive value for improved survival in the bevacizumab plus chemotherapy arm. Thus, a simple qRT-PCR assay of these four miRNAs may help identifying PM patients most likely to benefit from bevacizumab.

## Highlights

• Angiogenesis-related miRNAs are prognostic and predictive biomarkers in PM therapy.
• Low miR-193b-3p predicts longer survival in PM patient.
• Four miRNAs low expression boosts bevacizumab combo efficacy in MAPS trial.
• miRNA qRT-PCR assay may guide treatment selection for PM patients.

## Graphical abstract

**Figure:**
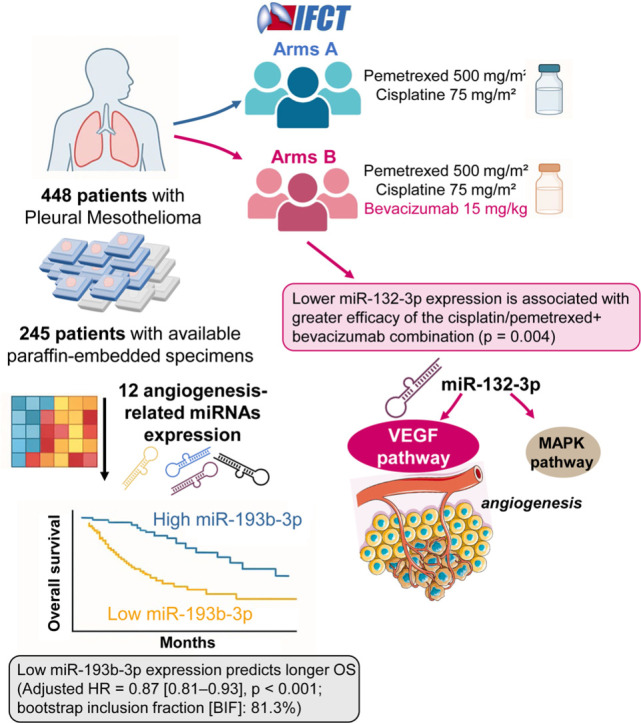

## Introduction

Pleural mesothelioma (PM) is a rare but aggressive cancer with a poor prognosis, primarily caused by occupational exposure to asbestos fibers [1,2]. The Mesothelioma Avastin Cisplatin Pemetrexed Study (MAPS) demonstrated the benefit of adding bevacizumab (a full-length recombinant humanized monoclonal antibody targeting VEGF) to cisplatin/pemetrexed chemotherapy, improving both overall survival (OS) and progression-free survival (PFS) in 448 patients with pleural mesothelioma (PM) [3,4]. The median OS reached 18.8 months, a benchmark that remains unsurpassed despite recent advances in immunotherapy for PM. Unexpectedly, classical neoangiogenesis biomarkers such as serum VEGF concentration [3] or VEGFR2/CD34 expression [5], failed to predict the response of PM patients to bevacizumab. Therefore, alternative biomarkers are needed to identify patients who may derive a survival benefit from bevacizumab-based therapy. Among potential candidates are microRNAs (miRNAs), small non-coding RNA molecules encoded by the eukaryotic genome. By repressing translation or promoting degradation of their target mRNAs, miRNAs regulate key cellular processes, including proliferation, growth, metabolism, and apoptosis [6]. Therefore, dysregulation of miRNA expression (either gain or loss) contributes to cancer development, including pleural mesothelioma (PM) (for reviews: [7,8]), and plays a role in key tumor processes such as neoangiogenesis [9], with such miRNAs often referred to as "angio-miRNAs". The expression of certain miRNAs is already known to predict the response to anti-angiogenic treatments in cancers such as colorectal cancer and glioblastoma. In ovarian cancer, studies have identified miRNAs as predictors of response to anti-angiogenic therapy by comparing their expression levels in responders versus non-responders using surgical samples from the primary tumor, collected before or after treatment [[10], [11], [12], [13]]. Therefore, microRNAs may have both diagnostic and prognostic potential in PM patients, as differences in miRNA expression between mesothelioma and normal mesothelial cells have already been demonstrated [14] (for review: [15]). Such molecules could serve routine theranostic purposes, as microRNAs are stable (unlike mRNAs) and easily measurable by qRT-PCR from routine fresh or archived formalin-fixed paraffin-embedded (FFPE) diagnostic samples, even of small size.

## Materials and methods

### Study design and participants

From February 13th, 2008, to January 5th, 2014, 448 patients were randomly assigned 1:1 to one of two treatments (223 to pemetrexed plus cisplatin and bevacizumab and 225 [50 %] to pemetrexed plus cisplatin) (Fig.S1). The formalin-fixed paraffin-embedded (FFPE) specimens were available for 245 patients (54,7 %, Fig.S1). Specific informed consent was obtained for the biological studies (Bio-MAPS), and the trial was approved by the appropriate ethics committee (CPP Ref 2007–20 Nord-Ouest III, France) with clinical main results previously reported elsewhere [3].

### MicroRNA assay

The miRNAs were extracted from the FFPE surgically resected tumor specimens containing at least 70 % of tumor cells using the miRNAeasy-FFPE kit (Qiagen™) according to the respective manufacturer’s instructions. The miRNA concentration was measured with the NanoDrop 2000 spectrophotometer (Thermo Scientific). The absorbance ratios (A230/A260 and A260/A280) were systematically assessed to verify the quality of the miRNA solution and to confirm the absence of contamination by solvents or proteins. These ratios were required to be between 1.8 and 2.0 for the quality to be considered sufficient for further analysis. If the ratios fell outside this range, the extraction and/or purification process was repeated.

Five ng of miRNAs were next retrotranscribed and amplified (PCR) using the TaqMan MiRNA Reverse transcription kit (Applied Biosystem) and the following Taq-Man MiRNA probes (Applied Biosystem), i.e. 20 microRNAs linked to tumor angiogenesis identified by literature curation and databases analysis with altered expression in PM: has-miR-15a-5p (ID000389), has-miR-15b-5p (ID000390), has-miR-21–5p (ID000397), has-miR-29c-5p (ID000415), has-miR-34c-5p (ID000428), has-miR-100–5p (ID000437), has-miR-126–5p (ID000450), has-miR-126–3p (ID000451), has-mir-132–3p (ID000457), has-miR-141–5p (ID000463), has-miR-155–5p (ID000479), has-miR-193a-3p (ID002250), has-miR-193b-3p (ID002367), has-miR-200a-3p (ID000502), has-miR-200b-3p (ID002251), has-miR-200c-3p (ID002300), has-miR-205–5p (ID000509), has-miR-210–3p (ID000512), has-miR-424–5p (ID000604) and has-miR-486–5p (ID001278) (Fig.S2). Each measurement was performed in duplicate, and the value retained for a given data point was the average of the two measurements, which were required not to differ by >1 Ct. A Ct value greater than 36 was considered indicative of no expression of the miRNA studied in the analyzed sample.

We then selected candidate miRNA biomarkers with a potential prognostic effect on overall survival (*p* < 0.2), using Cox models with Bonferroni-Holm correction leading to the identification of 12 miRNAs, for which data were available in 38 to 51 % of patients' samples (Fig.S2).

The RT-PCR data were normalized to the small nucleolar house-keeping RNA RNU48 (SNORD48) (assay ID 001,006). The average expression of RNU48 across all analyzed samples was 25.67 ± 0.15 Ct. Samples with an RNU48 Ct value greater than 30 were considered to have insufficient quality for miRNA quantification.

Positive standards and reaction mixtures lacking the reverse transcriptase were used routinely as controls for each miRNA sample. Relative quantification was conducted using the deltaCt method, where deltaCt is CtmiRX-CtRNU48.

### Statistical analysis

The Bio-MAPS study was a pre-specified ancillary and exploratory analysis. Baseline characteristics of patients with and without available microRNA profiling were compared using chi-squared tests for categorical variables, Student’s *t*-tests for continuous variables, and log-rank tests for time-to-event outcomes. Overall survival (OS) and progression-free survival (PFS) were estimated using the Kaplan–Meier method.

Univariate Cox proportional hazards models were first applied to assess the prognostic effect of each of the twelve tumor microRNAs (miRNAs) on progression-free survival (PFS) and overall survival (OS). To control for multiple testing and preserve the type I error rate, p-values were adjusted using the Bonferroni-Holm correction. MiRNAs with corrected p-values < 0.05 were subsequently entered into a stepwise multivariate Cox regression to identify an independent prognostic signature. The model included minimization factors used in the MAPS trial—histology, performance status (PS), and smoking status—as well as established clinical prognostic variables (sex, age, hemoglobin level, white blood cell count, and platelet count), which were forced into the model to estimate the adjusted prognostic impact of the selected biomarkers.

To evaluate the predictive value of individual miRNAs for treatment effect (pemetrexed-cisplatin with or without bevacizumab), a two-step approach was implemented, as proposed by [16]. First, miRNAs without a significant prognostic association with survival—after adjustment for treatment arm—were excluded. In the second step, for miRNAs passing this screening, interaction terms between treatment arm and miRNA expression were tested in multivariate Cox models adjusted for minimization variables. Bonferroni-Holm corrections were applied to interaction p-values.

Model robustness was assessed via bootstrap resampling (1000 iterations), and optimism-corrected concordance indices (c-index) were calculated to evaluate predictive performance. Statistical significance was defined as *p* < 0.05. All analyses were conducted using IBM SPSS Statistics, Version 22.0.

## Results

### Expression of angioMirs in PM patients from MAPS trial

Tumor cell content was insufficient (<70 %) to allow microRNA extraction in 240 of the 448 FFPE specimens (53.16 %). In addition, 2 samples (0.4 %) yielded low-quality RNA, preventing subsequent amplification (Fig.S1). For 2 out of the 238 remaining patients, miRNA quantification failed due to poor RNA quality. The expression of the twelve microRNAs was successfully assayed in 236 out of 448 patients (50.8 %). However, the number of successfully analyzed samples was lower for specific miRNAs: miR-132–3p (171/448), miR-424–5p (209/448), miR-100–5p (210/448), miR-210–3p (210/448), and miR-200b-3p (220/448). The baseline characteristics of the 236 patients with successful microRNA amplification (Fig. S1) did not differ significantly from those of patients with failed analyses (*n* = 212, Supplementary Table S1), including treatment arm allocation (with or without bevacizumab), histological subtype (epithelioid vs. non-epithelioid), age, performance status (0 vs. 1–2), and key biological prognostic variables in mesothelioma (hemoglobin concentration, leukocyte count, platelet count).

Establishing a correlation matrix, we found that the expression of the studied miRNAs was not correlated with that of angiogenesis markers previously analyzed in patient with PM from MAPS trial by immunohistochemistry, namely: CD34, VEGFR2 [5], VE-statin, IGFR1, YAP/TAZ and Amphiregulin (AREG) [17]. However, the expression levels of several miRNAs were correlated with each other, including miR-15a/miR-126* (0.86), miR-15a/miR-21 (0.93), miR-126/miR-126* (0.92), miR-126/miR-15a (0.82), miR-132/miR-15a (0.91), miR-424/miR-126* (0.83), miR-424/miR-21 (0.91), miR-424/miR-15a (0.96), miR-424/miR-132 (0.90), miR-15b/miR-126* (0.81), miR-15b/miR-15a (0.90), miR-15b/miR-424 (0.90), miR-193a/miR-193b (0.90), miR-193a/miR-15a (0.90), miR-193a/miR-100 (0.81), and miR-193a/miR-424 (0.84) (Fig.S3).

### Has-mir-193b-3p has a prognostic value for OS in PM patients from MAPS trial

**Fig. 1:**
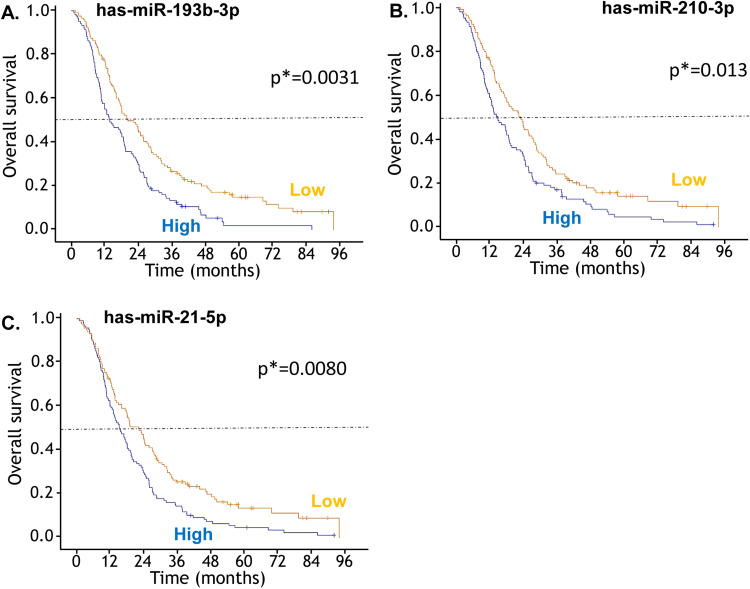
**Overall survival for the 236 patients with PM in the 'MAPS', NCT00651456 phase 3 trial, according to microRNA expression (A: has-miR-193b-3p, B: has-miR-210–3p, C: has-miR-21–5p).** Survival plots using cut-point at the median value. p*: Bonferroni-Holm corrected p-value for the continuous predictor in the Cox model.

In univariate analyses, we identified three miRs (has-mir-193b-3p, −210–3p and −21–5p) associated with PM patients OS (Table 1). Hazard Ratios, for a 1-point increase of the delta CT, were the following: mir-193b-3p: HR = 0.89, 95 %CI [0.84; 0.95], corrected *p* = 0.0031; miR210–3p: HR = 0.90, 95 %CI [0.84; 0.96], corrected *p* = 0.013; miR21–5p: HR = 0.90, 95 %CI [0.84; 0.96], corrected *p* = 0.0080. Survival plots using cut-points at the median value are presented on Fig. 1A-C (A: mir-193b-3p, B: miR210–3p, C: miR21–5p).

**Table 1:** Association of miRNAs with overall survival.

has-	n	HR*	IC95 %		p	corrected p†
**mir–**424–5p	209	0.95	(0.91;	0.99)	**0.019**	0.17
**mir–**155–5p	236	0.98	(0.94;	1.02)	0.26	0.78
**mir–**21–5p	236	0.90	(0.84;	0.96)	**0.00073**	**0.0080**
**mir-**193b −3p	236	0.89	(0.84;	0.95)	**0.00026**	**0.0031**
**mir-**200a −3p	236	0.99	(0.94;	1.05)	0.83	1
**mir-**210 −3p	210	0.90	(0.84;	0.96)	**0.0013**	**0.013**
**mir-**200b −3p	220	0.96	(0.91;	1.01)	0.12	0.59
**mir-**29c −5p	236	1.01	(0.96;	1.07)	0.72	1
**mir-**200c −3p	236	0.94	(0.88;	0.99)	**0.021**	0.17
**mir-**141 −5p	236	0.97	(0.94;	1.01)	0.14	0.58
**mir-**132 −3p	171	0.93	(0.87;	0.995)	**0.037**	0.26
**mir-**100 −5p	210	0.94	(0.88;	1.00)	0.063	0.38

⁎ For a 1-point increase of the ΔCT (CTmiR - CTRNU48).

† Bonferroni-Holm Method.

Only miR 193b-3p remained significant in the stepwise regression applied to the 3-miR model. The effect was still highly significant after adjustment on the minimization and clinical risk factors: adj. HR = 0.86, 95 %CI [0.80–0.92], *p* < 0.001, corrected c-index = 0.66. Median OS was 13.5 months 95 %CI (9.3 - 17.6) for patients with mir-193–3b high expression (> median value) and 22.3 months 95 %CI (16.5 - 28.0), for patients with mir-193–3b low expression.

Bootstrap resampling showed that, in univariate analyses, the same three miRNAs (has-mir-193b-3p, −210–3p and −21–5p) were the most frequently associated to survival (in respectively 81 %, 71 % and 68 % of the 1000 bootstrapped samples). After multivariate analysis, mir-193b-3p was the most frequently selected miRNA, but in only 42 % of the cases. Bootstrap inclusion fractions (BIF) were 21 % for mir-21–5p and 16 % for mir-210–3p

None of the twelve miRNAs was significantly associated to PM patients PFS in univariate analyses, after Bonferroni-Holm corrections. No multivariate model was thus estimated.

### Has-miR-132–3p has a predictive value for OS in PM patients from MAPS trial

To exclude non-prognostic microRNAs from subsequent predictive analyses, in the first step of the analysis, we identified seven microRNAs (miR-424, miR-21, miR-193b, miR-210, miR-200c, miR-132, and miR-100) that showed a significant association with OS, independently of treatment arm (beva+chemo arm as compared with those treated with only chemo).

**Fig. 2:**
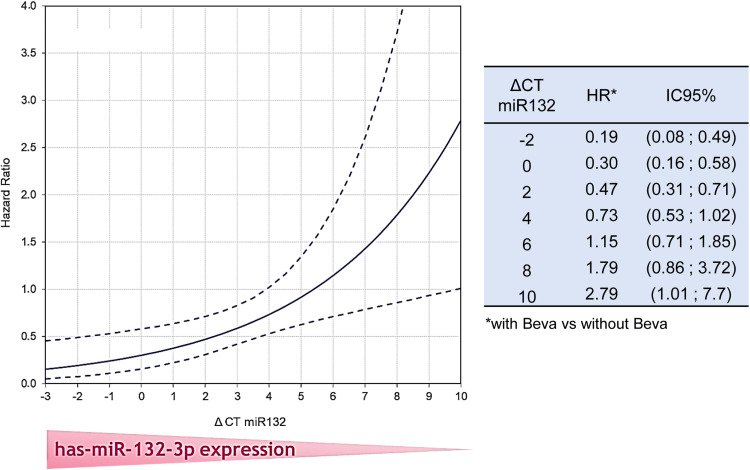
Variation of the bevacizumab effect on OS according to DeltaCT has-mir-132–3p.

In the first screening step, five microRNAs with poor prognostic value were excluded from the analysis: hsa-miR-155–5p, −200a-3p, −200b-3p, −29c-5p, and −141–5p In the second step, among the seven remaining biomarkers, only miR-132–3p demonstrated a significant predictive value after Bonferroni-Holm correction (Table 2). The variation of the bevacizumab effect, according to mir-132–3p expression, is illustrated on Fig. 2: the higher the expression level of miR-132, the better the efficacy of the pemetrexed-cisplatin-bevacizumab triplet. Indeed, patients with high tumor miR-132 expression (> median value) had a median overall survival (OS) of 17.1 months (95 % CI: 8.5–25.8) in the bevacizumab arm versus 11.2 months (95 % CI: 8.9–13.6) in the chemotherapy-alone arm. Conversely, for patients with low tumor miR-132 expression, median OS was 24.6 months (95 % CI: 19.1–30.1) in the bevacizumab arm versus 24.7 months (95 % CI: 20.1–29.3) in the chemotherapy-alone arm.

**Table 2:** Interaction effect of miRNAs on overall survival.

has-	n	p*	corrected p†
**mir–**424–5p	209	0.13	0.50
**mir–**21–5p	236	0.10	0.50
**mir-**193b −3p	236	0.48	0.95
**mir-**210 −3p	210	0.71	0.95
**mir-**200c −3p	236	0.10	0.50
**mir-**132 −3p	171	**0.0043**	**0.030**
**mir-**100 −5p	210	**0.036**	0.21

⁎ Interaction between treatment arm and miRNA expression in a Cox model adjusting for the minimization factors.

† Bonferroni-Holm Method.

Bootstrap resampling indicated that the predictive effect was most frequently detected for miR-132–3p, with a significant interaction p-value observed in 40 % of 1000 bootstrapped samples. The next biomarkers in the ranking were miR-100–5p (BIF 19 %) and miR-21–5p (BIF 18 %). No statistically significant interaction effect was observed for PFS.

## Discussion

Out of 20 angiogenesis-related microRNAs identified by literature curation and databases analysis with altered expression in PM, twelve microRNA were retained in the current study following an intermediate statistical analysis step to identify candidate miRNAs that would help in identifying PM patients likely to specifically benefit from antiangiogenic treatment bevacizumab in combination with Cis/pemetrexed.

First, since transcriptional co-regulation is frequently reported for multiple miRNAs whose genes are often clustered in common regions of the human genome, we investigated whether the expression of some of these 12 selected miRNAs could be correlated with each other. We found that the expression of the studied miRNAs was not correlated with other angiogenic biomarkers analyzed in MAPS [5,17], whereas the expression levels of several miRNAs were correlated with each other. This suggests that microRNAs involved in the same biological process—such as angiogenesis—have correlated expression profiles, which is consistent with the findings of [18]. These authors demonstrated that several microRNAs, including miR-221 and other angiogenic miRNAs, are co-expressed and correlated during the formation of endothelial tip cells, a key process in angiogenesis. These miRNAs act as a network jointly regulating shared biological pathways [18].

Although we cannot exclude potential biases due to sample attrition in our miR study, which was successfully conducted in only half of the initial clinical trial population, we found no evidence of disproportionate sample loss from specific patient subgroups. No statistical differences were observed between the subsets with or without miR analyses in terms of patient characteristics, and both subsets showed similar median PFS and OS values (Table S1).

Considering this potential limitation, we found that the expression of hsa-miR-193b-3p, −210–3p, and −21–5p microRNAs was associated with prognosis in pleural mesothelioma patients from the MAPS trial (both treatment arms combined), using stratified univariate Cox models. However, only one miRNA, hsa-miR-193b-3p, retained a prognostic effect in the multivariate model that included all classical clinical and pathological prognostic variables as well as stratification factors, after Bonferroni correction for multiple testing and bootstrap validation. There are few data in the literature regarding the pathophysiological roles of hsa-miR-193b-3p, but the available evidence suggests it may act as a tumor suppressor in ovarian cancer, lung cancer, and leukemia [[19], [20], [21], [22]]. Thus, the finding that low expression of hsa-miR-193b-3p is associated with longer survival in PM patients in our study initially appeared surprising. However, other studies have also suggested that hsa-miR-193b-3p may have a controversial and/or histology-dependent role. In colorectal cancer, for example, hsa-miR-193b-3p has been shown to act as an oncomiRNA rather than exhibiting tumor-suppressive properties [23]. hsa-miR-193b-3p can suppress cell proliferation, inflammatory cytokine secretion, and the STAT3 [[24], [25]] and NF-κB signaling pathways [[26], [27], [28]]. In pleural mesothelioma, the NF-κB pathway is known to promote the survival of mesothelial cells [29,30]. Other data that which may help reconcile our findings suggesting a potential oncogenic role for hsa-miR-193b-3p in PM show that miR-193b induces EMT change and migration ability through the regulation of TGFβ2 expression in A549 cells [31]. In addition, miR193b-3p high expression has also been associated with lower cancer cell differentiation, higher TNM stage and lymph node metastases in non-small cell lung cancer, by targeting TP53 pathway, cell-cell adhesion, cytoskeleton remodeling or autophagy (AKT3, IGF-1R), underlining the complex role of such miR [32]. However, further investigation is required to draw a definitive conclusion.

We report that seven microRNAs may have predictive value for overall survival (OS) in patients specifically treated with a bevacizumab-based triplet, compared to those receiving chemotherapy alone, as shown by univariate Cox models with interaction tests. Among them, only one microRNA, miR-132–3p, retained its predictive value in the multivariate analysis after Bonferroni correction. It is known that the power of interaction tests, performed as a secondary objective in a randomized trial, is significantly reduced compared to the power defined for the overall effect. This reduction is further amplified, in our study, by the application of Bonferroni corrections. We therefore cannot exclude that the power was not sufficient to detect the predictive effect of other miRNAs.

MiR-132–3p is known to have pleiotropic targets [33,34]. The predictive impact observed in our study may be explained by the role of miR-132–3p in targeting the VEGF pathway. Indeed, in endothelial cells and pericytes, miR-132–3p is involved in a VEGF signaling loop induced by hypoxia [35], and it also regulates microvessel density and cell proliferation [36]. However, other microRNAs targeting the VEGF pathway did not show any independent predictive effect. Furthermore, we previously reported that neither plasma VEGF levels [3], nor VEGFR2 immunostaining, nor the CD34 endothelial cell marker demonstrated any predictive value in MAPS patients [5]. A speculative explanation could be that miR-132–3p exerts a unique effect on vascular tubulogenesis and vessel density regulation [35], which may not be influenced by other microRNAs. Experimental data are currently awaited to support this hypothesis.

The predictive value of miR-132–3p expression should be validated in an independent cohort of randomized patients comparing bevacizumab (or another anti-angiogenic agent) with a control arm. Such findings would warrant experimental studies using an antagomiR to knock down miR-132–3p, thereby disrupting the VEGF/VEGFR2 autocrine loop in PM cells.

Given the current paradigm shift in treatment toward immune checkpoint inhibitors (ICIs), both in the second-line setting [37] and increasingly in first-line treatment [38], miR-132–3p expression also merits investigation in cohorts of patients treated with ICIs, with or without bevacizumab—such as in the ETOP BEAT‑*meso* trial [39]. Although the BEAT‑*meso* trial yielded negative statistical results, the experimental arm combining atezolizumab and bevacizumab demonstrated numerically longer survival than the control arm. It is worth exploring whether imbalances in miR-132–3p expression might have masked the overall survival benefit of the ICI–bevacizumab combination. This question is especially relevant in light of the development of bispecific antibodies targeting both PD-1 and VEGF, such as ivonescimab. Additionally, miR-132–3p expression should be studied in patient cohorts from trials comparing immune checkpoint inhibitor (ICI) combinations—such as the CheckMate-743 trial [38] (dual ICI) and the IND-227 trial [40] (ICI plus chemotherapy)—against chemotherapy alone. This is particularly important because VEGF pathway regulation is known to influence immune responses, not only by modulating vascular permeability to immune cells [41,42], but also by promoting an immunosuppressive tumor microenvironment [43,44]. Notably, miR-132–3p regulates the expression of HB-EGF, a cytokine produced by monocytes and macrophages that may play a role in immune response and cancer progression [45]. Furthermore, another miR-132–3p target, the SOX4 transcription factor, has been implicated in T-cell differentiation and immune evasion [46]. Collectively, these considerations underscore the need for further studies of miR-132–3p expression in patients with pleural mesothelioma treated with ICIs and VEGF inhibitors. Relevantly, it is well established that miRNAs can modulate the response to ICIs by regulating key immune pathways involved in tumor immune evasion and response [47,48]. Moreover, miRNAs have the advantage of being measurable in blood, offering a minimally invasive means to monitor patients during therapy [49]. Importantly, circulating miRNAs often reflect the molecular characteristics of the tumor cells that produce them, thereby serving as potential real-time surrogates of tumor biology and therapeutic response [50].

## Clinical trial information

Specific informed consent was obtained for the biological studies (Bio-MAPS). and the trial was approved by the appropriate ethics committee (CPP Ref 2007–20 Nord-Ouest III. France). All data are stored at the IFCT center and can be made available upon request.

## Prior presentation

Presented as oral presentation at the IMIG Congress 2023, Lille, France, June 26, 2023.

## Funding sources

Franck Morin reports financial support was provided by Roche-France (2013). Solenn Brosseau reports financial support was provided by CHU de Caen (APRI 2012). Gerard Zalcman reports financial support was provided by Ligue contre le Cancer de Normandie (2012). Guenaelle Levallet reports financial support was provided by Fond de Recherche en Santé Respiratoire (FRSR 2012).

## CRediT authorship contribution statement

**Guénaëlle Levallet:** Writing – review & editing, Writing – original draft, Visualization, Validation, Supervision, Resources, Project administration, Methodology, Investigation, Funding acquisition, Formal analysis, Data curation, Conceptualization. **Christian Creveuil:** Writing – review & editing, Writing – original draft, Visualization, Validation, Software, Methodology, Formal analysis, Data curation. **Alexandre Léger-Vigot:** Writing – review & editing, Writing – original draft, Visualization, Validation, Methodology, Formal analysis, Data curation. **Solenn Brosseau:** Writing – review & editing, Writing – original draft, Visualization, Validation, Investigation, Funding acquisition, Formal analysis, Data curation. **Claire Danel:** Writing – review & editing, Writing – original draft, Visualization, Validation, Investigation, Data curation. **Arnaud Scherpereel:** Writing – review & editing, Writing – original draft, Visualization, Validation, Investigation, Data curation. **Sylvie Lantuejoul:** Writing – review & editing, Writing – original draft, Visualization, Validation, Investigation, Data curation. **Julien Mazières:** Writing – review & editing, Writing – original draft, Visualization, Validation, Investigation, Data curation. **Laurent Greillier:** Writing – review & editing, Writing – original draft, Visualization, Validation, Investigation, Data curation. **Clarisse Audigier-Valette:** Writing – review & editing, Writing – original draft, Visualization, Validation, Investigation, Data curation. **Emmanuel Bergot:** Writing – review & editing, Writing – original draft, Visualization, Validation, Investigation, Data curation. **Denis Moro-Sibilot:** Writing – review & editing, Writing – original draft, Visualization, Validation, Investigation, Data curation. **Olivier Molinier:** Writing – review & editing, Writing – original draft, Visualization, Validation, Investigation, Data curation. **Hervé Léna:** Writing – review & editing, Writing – original draft, Visualization, Validation, Investigation, Data curation. **Isabelle Monnet:** Writing – review & editing, Writing – original draft, Visualization, Validation, Investigation, Data curation. **Franck Morin:** Writing – review & editing, Writing – original draft, Visualization, Validation, Resources, Project administration, Funding acquisition. **Valérie Gounant:** Writing – review & editing, Writing – original draft, Visualization, Validation, Investigation. **Gérard Zalcman:** Writing – review & editing, Writing – original draft, Visualization, Validation, Supervision, Resources, Project administration, Methodology, Investigation, Funding acquisition, Formal analysis, Data curation, Conceptualization.

## Declaration of competing interest

The authors declare no conflicts of interest related to the data presented in this article.

## Appendix Supplementary materials

Image, application 1

Image, application 2

Image, application 3

Image, application 4
