# Supplementary material for: MiR-193b-3p and miR-132-3p as prognostic biomarkers of survival in pleural mesothelioma patients treated with first-line bevacizumab plus pemetrexed-platinum chemotherapy in the IFCT-0701 MAPS phase 3 trial
Source: Transl Oncol. 2025 Sep 5;61:102520. doi: 10.1016/j.tranon.2025.102520 (PMC12447922; doi:10.1016/j.tranon.2025.102520)
Supplement: Supplementary file 1 [file mmc1.pptx]

## Slide 1
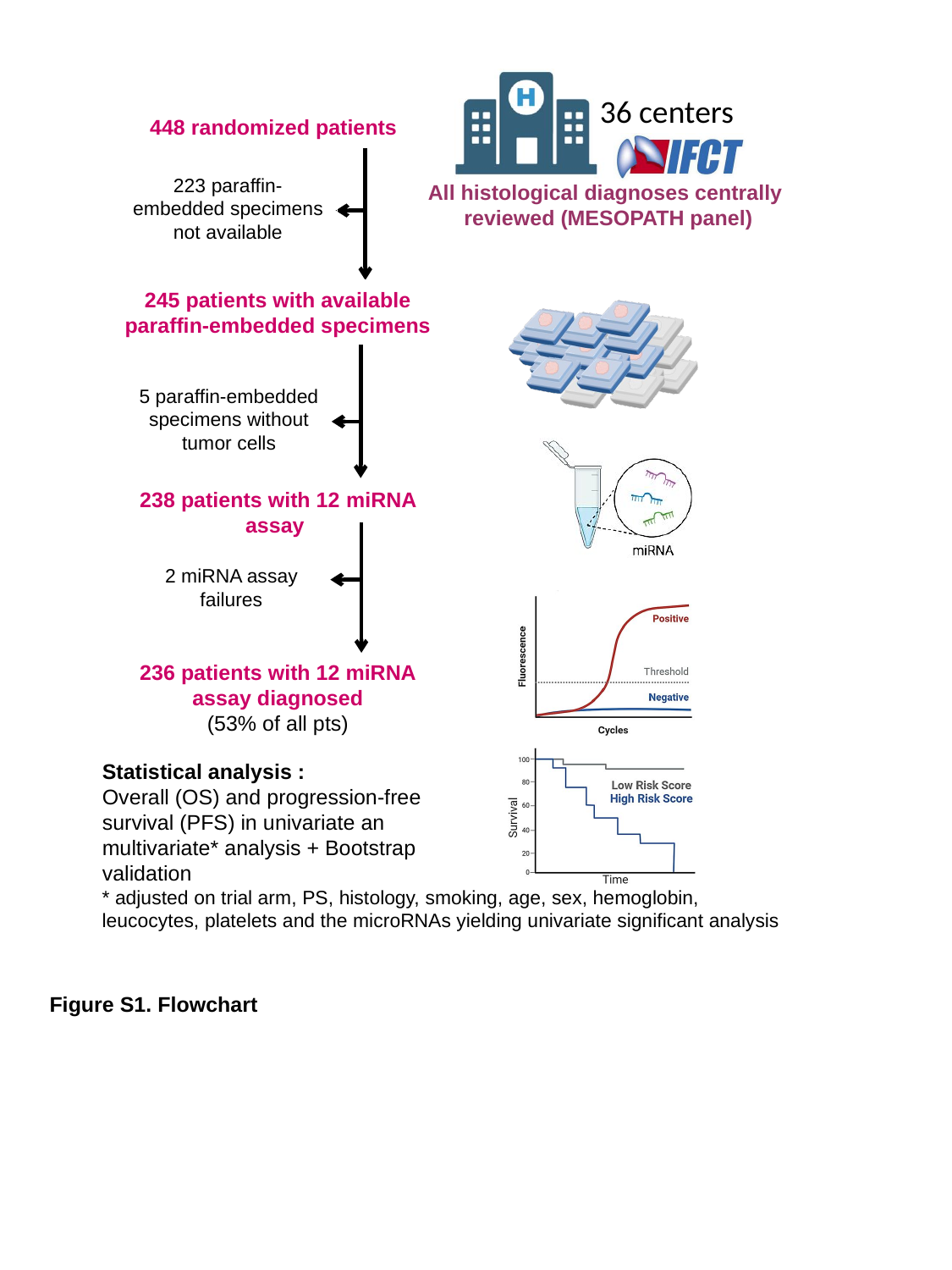

36 centers
448 randomized patients
223 paraffin-embedded specimens not available
All histological diagnoses centrally
 reviewed (MESOPATH panel)
245 patients with available paraffin-embedded specimens
5 paraffin-embedded specimens without tumor cells
238 patients with 12 miRNA assay
2 miRNA assay failures
236 patients with 12 miRNA assay diagnosed
(53% of all pts)
Statistical analysis :
Overall (OS) and progression-free survival (PFS) in univariate an multivariate* analysis + Bootstrap validation
* adjusted on trial arm, PS, histology, smoking, age, sex, hemoglobin, leucocytes, platelets and the microRNAs yielding univariate significant analysis
Figure S1. Flowchart
