# Supplementary material for: MiR-193b-3p and miR-132-3p as prognostic biomarkers of survival in pleural mesothelioma patients treated with first-line bevacizumab plus pemetrexed-platinum chemotherapy in the IFCT-0701 MAPS phase 3 trial
Source: Transl Oncol. 2025 Sep 5;61:102520. doi: 10.1016/j.tranon.2025.102520 (PMC12447922; doi:10.1016/j.tranon.2025.102520)
Supplement: Supplementary file 2 [file mmc2.pptx]

## Slide 1
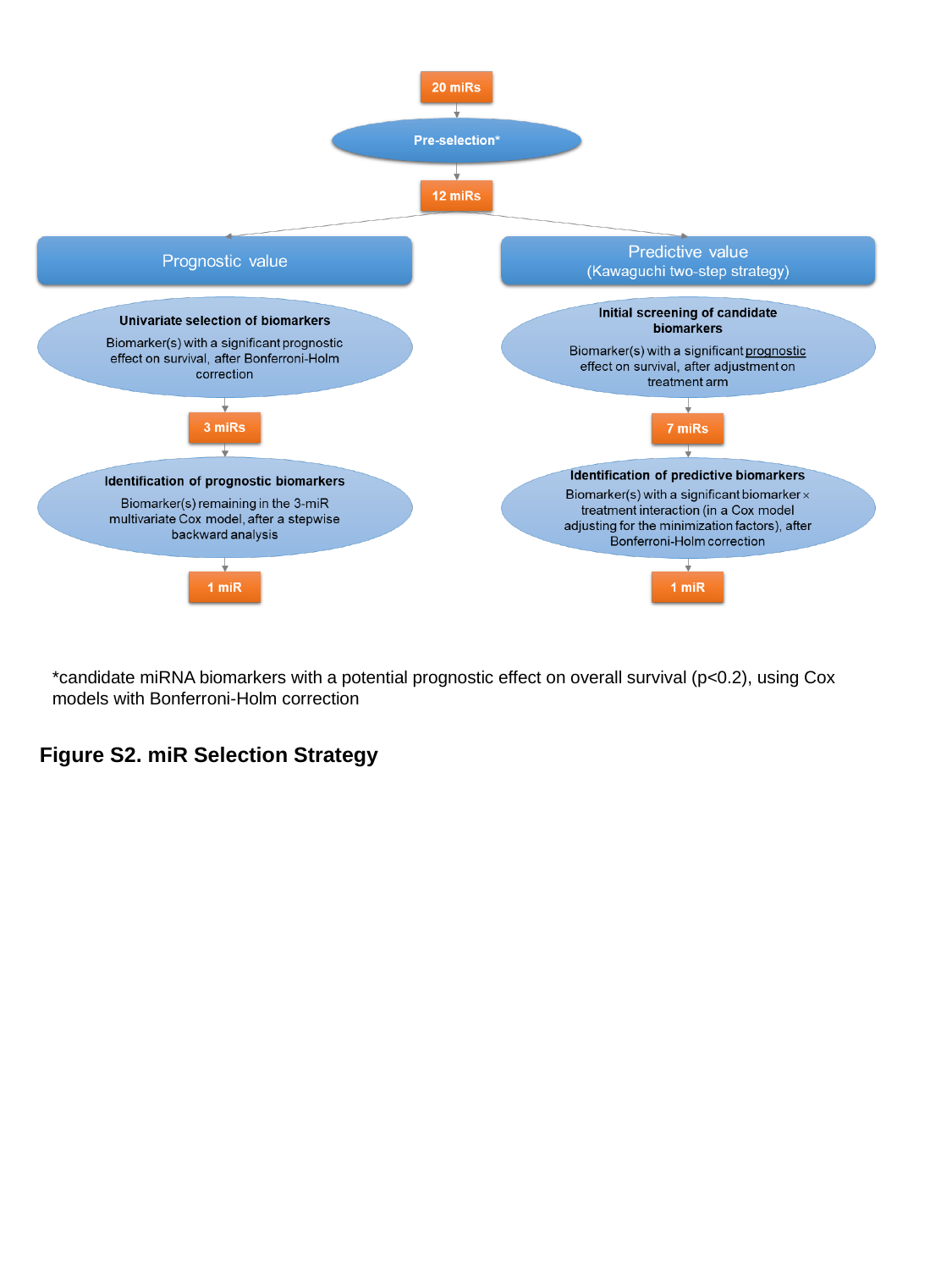

*candidate miRNA biomarkers with a potential prognostic effect on overall survival (p<0.2), using Cox models with Bonferroni-Holm correction
Figure S2. miR Selection Strategy
