# Supplementary material for: MiR-193b-3p and miR-132-3p as prognostic biomarkers of survival in pleural mesothelioma patients treated with first-line bevacizumab plus pemetrexed-platinum chemotherapy in the IFCT-0701 MAPS phase 3 trial
Source: Transl Oncol. 2025 Sep 5;61:102520. doi: 10.1016/j.tranon.2025.102520 (PMC12447922; doi:10.1016/j.tranon.2025.102520)
Supplement: Supplementary file 3 [file mmc3.pptx]

## Slide 1
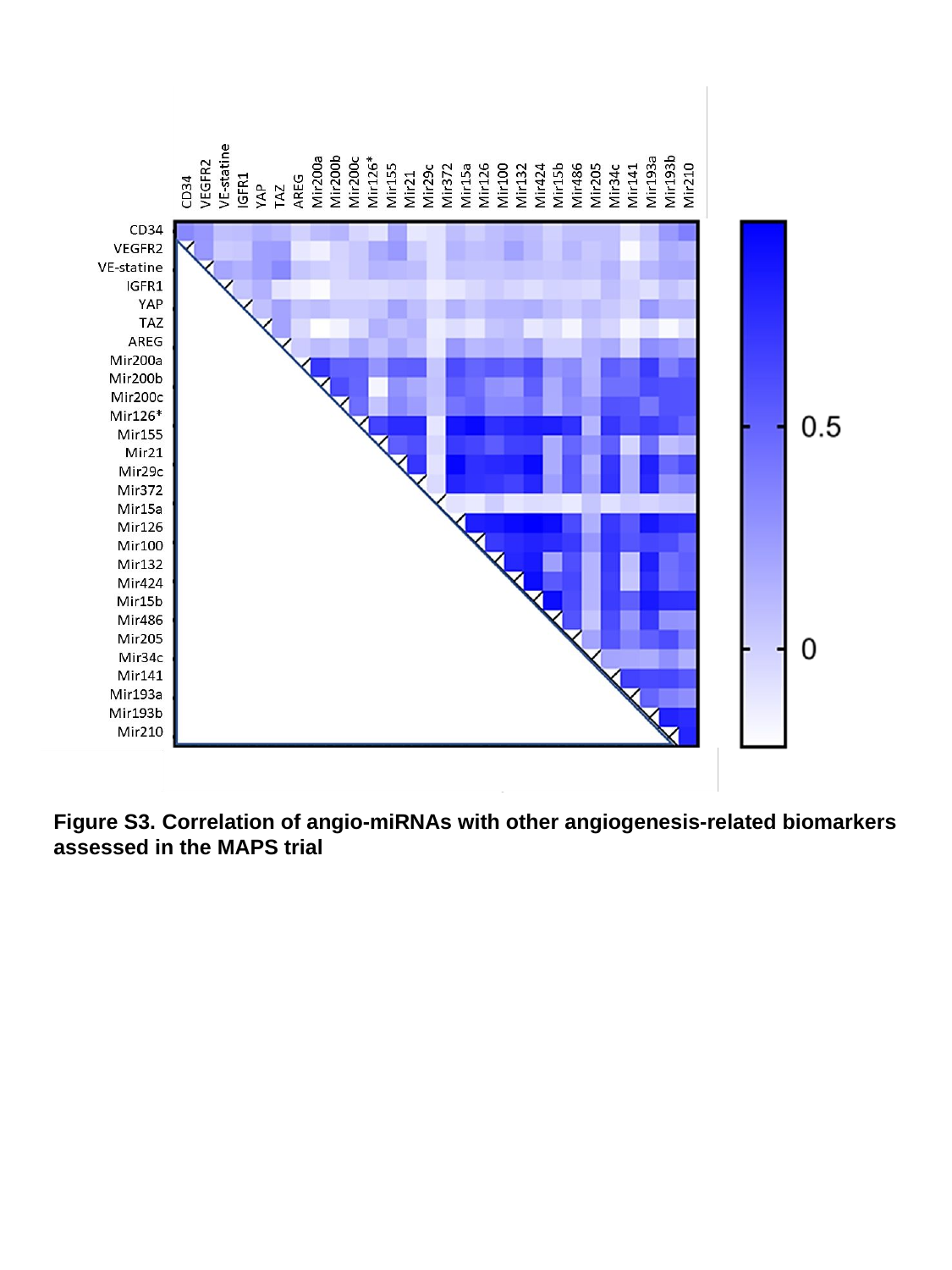

Figure S3. Correlation of angio-miRNAs with other angiogenesis-related biomarkers assessed in the MAPS trial
