# Supplementary material for: MiR-193b-3p and miR-132-3p as prognostic biomarkers of survival in pleural mesothelioma patients treated with first-line bevacizumab plus pemetrexed-platinum chemotherapy in the IFCT-0701 MAPS phase 3 trial
Source: Transl Oncol. 2025 Sep 5;61:102520. doi: 10.1016/j.tranon.2025.102520 (PMC12447922; doi:10.1016/j.tranon.2025.102520)
Supplement: Supplementary file 4 [file mmc4.docx]

**TableS1*.*** **Characteristics of Bio-MAPS (patients with available microRNA MSP-PCR) and MAPS patients without MicroRNA analysis.**

|  | | Bio-MAPS (n = 236) | | MAPS (n = 212) | | p* |
| --- | --- | --- | --- | --- | --- | --- |
|  |  | n | % | n | % |  |
| Group | CT | 116 | 49.2% | 109 | 51.4% | 0.63 |
|  | CT+Beva | 120 | 50.8% | 103 | 48.6% |  |
| Age at inclusion [m(sd)] | | 65.1 | 7.3 | 64.9 | 7.1 | 0.80 |
| Sex | men | 177 | 75.0% | 161 | 75.9% | 0.82 |
|  | women | 59 | 25.0% | 51 | 24.1% |  |
| PS | 0-1 | 228 | 96.6% | 205 | 96.7% | 0.96 |
|  | 2 | 8 | 3.4% | 7 | 3.3% |  |
| Smoking | Never smokers | 98 | 41.5% | 96 | 45.3% | 0.42 |
|  | smokers | 138 | 58.5% | 116 | 54.7% |  |
| Histology | epithelioïd | 191 | 80.9% | 172 | 81.1% | 0.97 |
|  | sarcomatoïde/ biphasic | 45 | 19.1% | 40 | 18.9% |  |
| leukocytes [m (sd)] | | 8.7 | 2.7 | 8.3 | 2.5 | 0.13 |
| Hemoglobin [m (sd)] | | 13.2 | 1.7 | 13.2 | 1.5 | 0.77 |
| Platelets [median (IQR)] | | 318 | 263 - 412 | 307 | 247 - 389 | 0.10 |
| PFS [median (months)] | | 236 | 8.5 | 212 | 8.0 | 0.42 |
| OS [median (months)] | | 236 | 17.9 | 212 | 16.7 | 0.94 |
| *Khi² test for qualitative data. Student t-test for continuous data. Logrank test for survival data | | | | | | |

PCB=pemetrexed plus cisplatin plus bevacizumab. PC=pemetrexed pluscisplatin. PFS=Progression-free survival. OS=Overall survival.
